# Continuous venovenous hemofiltration decreases mortality and ameliorates acute lung injury in canine model of severe salt water drowning

**DOI:** 10.1186/s13049-016-0224-5

**Published:** 2016-03-31

**Authors:** Jian Chen, Guangming Chen, Daping Xiao, Weihua Peng, Guoqing Yu, Yueyong Lin, Feng Zheng

**Affiliations:** Department of Nephrology, Fuzhou General Hospital, Nanjing Military Command, Fuzhou, China; Department of Nephrology, The Second Hospital, and Advanced Institute for Medical Sciences, Dalian Medical University, Dalian, 116044 China

**Keywords:** Drowning, Hemofiltration, Pulmonary injury

## Abstract

**Background:**

Pulmonary edema is an important cause of complications and death in severe drowning. Continuous veno-venous hemofiltration (CVVH) may reduce pulmonary edema and thus may be a treatment modality for severe sea water drowning resuscitation.

**Methos:**

20 dogs were anesthetized and tracheally intubated. 10 ml/kg of sea water was infused into trachea in a minute. All animals developed signs of respiratory distress and severe hypoxia (PaO_2_ < 40 mmHg) within 15 minutes after infusion. They were then mechanical ventilated and randomized to receive either CVVH (*n* = 10) or no additional treatment (control, *n* = 10) and followed over 4 hours. Arterial gas, hemodynamic parameters, and the levels of circulating inflammatory cytokines including interleukin 6 (IL-6), interleukin 8 (IL-8), and tumor necrosis factor α (TNFα) were determined. Additionally, blood endothelin and the levels of oxidative stress in lung were measured at sacrifice.

**Results:**

5 animals in the control group (50 %) died within 4 hours after sea water aspiration, while 10 animals received CVVH all survived (*p* < 0.05). Importantly, CVVH significantly improved blood gas exchange as evidenced by higher PaO_2,_ normal oxygen saturation, and no carbon dioxide retention after 3 hour of CVVH, while also correcting against acidosis. Levels of circulating IL-6, IL-8, and TNFα were elevated in control but not in CVVH group (*p* < 0.01). CVVH also reduced plasma endothelin and alleviated oxidative stress. Histology examination further revealed reductions in pulmonary alveolar injury, blood congestion, and inflammation by CVVH.

**Discussion and conclusions:**

CVVH decreased mortality and pulmonary injury and largely maintained hemodynamic and acid-base balance in animals with severe sea water drowning and thus, may be added as a new measure to aid in resuscitation from severe sea water drowning.

**Trial registration:**

Animal protocol number: FZG0001859 http://www.fzzyy.com.

## Background

Drowning is one of the leading causes of accidental death. Drowning-induced respiratory failure can cause severe organ damage through hypoxia, as airway obstruction decreases oxygen inspiration. The presence of excessive water in lung alveoli greatly impedes blood-gas exchange, resulting in decreased arterial partial oxygen pressure (PaO_2_) and excess carbon dioxide retention [1, 2]. While these obstructions occur in drowning cases irrespective of the medium, the pulmonary edema that occurs may be especially exacerbated in cases of salt-water drowning, as the intake of hypertonic fluids pulls water out from the blood. This phenomenon has been previously documented in animals receiving intratracheal seawater, which caused multi-fold increases in the wet weight of the lungs far above the amount of aspirate [3]. Pulmonary edema also occurs after fresh drowning due to alveolar injury that resulted in increased epithelial permeability and secondary inflammation and cardiogenic factors. As such, removal of airway obstruction, via removal of excessive fluid in alveoli and restoration of normal blood-gas exchange are critical for resuscitation from drowning [4].

Hemodialysis that filters out water and small molecules from blood and eventually from interstitial space via diffusion and/or convection is a common replacement therapy for kidney failure. Since volume overload and other cause of cardiogenic pulmonary edema are usually the result of increased hydrostatic pressure, which drives fluid into alveoli, the timely reduction of hydrostatic pressure by hemodialysis offers an effective intervention possibility. At the same time, hemodialysis has also been suggested to be potentially useful for cases of non-cardiogenic pulmonary edemas. While non-cardiogenic pulmonary edema is typically caused by increased permeability in lung blood vessel endothelial and alveolar epithelial barriers, leading to leakage of serum constituents into alveoli, recent studies using continuous renal replacement therapy (CRRT) has also emerged as an important treatment modality in these cases. Continuous venovenous hemofiltration (CVVH) is one of CRRT techniques that is applied extensively in intensive care units. CVVH has been shown to lead to improved hemodynamic stability, as well as steady acid-base and electrolytes corrections. Importantly, CVVH may also help to reduce acute inflammation by removing small pro-inflammatory cytokines and signaling molecules such as tumor necrosis factor α (TNFα) and various interleukins [5, 6]. This additional capability has caused CVVH to be used to help treat the non-cardiogenic acute respiratory distress that may occur in cases of sepsis. The influx of exogenous fluid into lung as in the case of severe drowning has similarly been shown to induce a broad inflammatory response [7]. The inflammation would likely be further heightened in sea water drowning due to high medium osmolality, a factor that has been shown to promote inflammation *in vitro*. Thus, CVVH may be applied to aid in resuscitation from severe sea water drowning for the purposes of both reducing pulmonary edema and decreasing inflammation. We tested this potential in this study using a canine model of involuntary sea water aspiration.

## Methods

### Animals and sea water drowning

20 age- and breed-matched local male dogs weighing 10–15 kg were used for the experiment. The number of animals, study protocol, and procedures were approved by animal care and usage committee at Fuzhou General Hospital. Animals were fasted for 8 hours before the experiment. General anesthesia was initiated and maintained by ketamine (20–40 mg/kg, intramuscularly). Animals were placed in the supine position and trachea intubation was performed. The right jugular vein, two femoral veins, and the right femoral artery were cannulated. Arterial blood pressure was monitored and blood gas was sampled from the right femoral artery. Central venous pressure was determined by placing a catheter in the abdominal vena cava via the femoral vein. A urinary catheter was inserted into bladder via the urethra. The sea water drowning model was established using a modified protocol from Modell et al. [8]. Briefly, 10 ml/kg of sea water obtained directly from the South China Sea (osmolarity 803.33 mmol/kg, Na^+^ 427 mM,K^+^ 9.4 mM, Mg^2+^ 16.25 mM,Cl^-^ 484 mM,Ca^2+^ 12.67 mM) was infused into animal trachea within a minute. Within 15 minutes after sea water aspiration, signs of respiratory distress including difficulty in breath and audible crackle were evident, and PaO_2_ had dropped to below 40 mmHg in all animals, confirming the establishment of the model. Mechanical ventilation was given to animals with a Bear respirator with an adjustable tidal volume at the maximal of 10 ml/kg, a pressure controlled ventilation of 25-30 cmH_2_O, a positive end-expiratory pressure of 8 cmH_2_O, an inspiratory fraction of oxygen 0.5, and a respiratory rate of 30 breaths/minute [9]. Animals were then randomly assigned to either simultaneously receive CVVH (*n* = 10) or no further treatment (*n* = 10). Animals were followed for 4 hours after sea water infusion and were sacrificed by given intravenous injection of overdose of anaesthetic and 10 ml of 10 % potassium chloride. Since the vital signs and the hemodynamic parameters were continually monitored during the experiment, humane end points were determined by two physicians if an animal had a 80 % reduction in heart rate and blood pressure for more than 10 minutes.

### Continue venovenous hemofiltration (CVVH)

A double lumen catheter was inserted into the external jugular vein. CVVH was performed with a Baxter CRRT machine. Since previous reports and our preliminary experiment showed that hypovolemia developed in animals after sea water drowning due likely to high salts in lungs that induce the loss of water from blood [9], hydroxyethyel starch and high salt were added to replacement solution (Osmolarity 335.1 mmol/kg,hydroxyethyel starch 1.6 %, Na^+^ 164.16 mM,Cl^-^124.98 mM, Ca^2+^ 2.71 mM,K^+^ 4.47 mM, HCO_3_^-^ 43.65 mM,Mg^2+^ 1.52 mM, Glu 13.89 mM). The replacement solution was delivered prior to filtration at the speed of 150 ml/kg/h. CVVH was accomplished using a PSHF (Polysulfone High-flux membrane) filter with 80 ml/min blood flow rate. Heparin was administrated as an anticoagulant at loading dose of 1250 IU/kg and maintenance dose of 625 IU/kg/h. CVVH was run for the entire 4 hours. In the first hour, a balance between output (ultrafiltration volume plus urine) and input (replacement solution delivered) was maintained. Thus, no extra fluid was removed, to prevent the worsening of hypovolemia. A gradual increase in fluid removal was then achieved by increasing ultrafiltration rate over the next three hours. By the end, the net amount of fluid removed by CVVH was matched to the volume of sea water (10 ml/kg) infused into the trachea.

### Plasma endothelin, IL-8, IL-6, and TNF-α

Blood samples were collected from animals at baseline and at 15, 120, and 240 minutes after seawater aspiration in EDTA coated tubes. Plasma was isolated via centrifugation at 3500 rpm for 5 minutes at 4 °C. Endothelin containing fractions were harvested via a C18 Sep-PaK column. Endothelin 1 (ET-1) levels were measured by enzyme immunometric assay with an anti-ET-1 antibody. The levels of IL-8, IL-6, and TNF-α were determined using ELISA kits following manufacturer’s protocols (R&D systems, Inc.).

### Lung

At sacrifice, namely 4 hours and 15 minutes after involuntary sea water aspiration, both lungs were collected and weighed. The levels of superoxide dismutase (SOD) and a key lipid oxidation product, malonaldehyde (MDA), were measured in lungs using colorimetric assay kits. Briefly, 100 mg of lung tissues were homogenized on ice and subsequently centrifuged at 12,000 g for 10 minutes (for SOD assay) or 10,000 g for 5 minutes (for MDA assay) at 4 °C. Supernantants were collected and protein concentration in suprenatant was quantitated. Samples were reacted with SOD or MDA assay reagents and the intensity of color generated by the reactions was recorded with a microplate reader. Known amounts of SOD or MDA standards was added to the reactions to obtain a standard curve for determining the concentration in each sample. The final result was corrected to the overall sample protein concentration. For pathology, pieces of tissues were fixed in 10 % formalin and processed for hematotoxylin and eosin staining. Slides were evaluated by an independent pathologist blinded to the experimental groups.

### Statistical analysis

The differences in animal survival rate between CVVH treated and control group were assessed using the χ2 test. Other values presented were expressed as mean ± 1SD. One-way analysis of variance or T test in SPSS 13.0 software was selected to test the differences between the groups and time points. *p* < 0.05 was regarded as statistically significant.

## Results

### Hemodynamic changes and survival

After initiation of the sea-water drowning model, all animals developed respiratory distress within 15 minutes of intratracheal infusion. All animals were provided mechanical ventilation support as described in the methods, and were subsequently monitored for several common characteristics. The degree of hypoxia and acidosis was comparable between the CVVH-treated and control groups at the outset, and hemodynamic changes such as bradycardia, hypotension, and elevated plasma osmolarity were also similar (Table 1). While there was a trend of increase in central venous pressure (CVP) in animals treated via CVVH, the values did not reach statistical difference compared to control. Heart beats were slower and blood pressure levels were significantly reduced in all animals after sea water aspiration. CVVH treatment did not improve slow heart beat but had largely corrected hypotension. Plasma osmolarity was increased after sea water drowning, and the increase was more pronounced in animals treated with CVVH, likely a result of the application of high- osmolarity replacement solution used in CVVH to balance the hypertonicity of sea water infused. This increase was not associated with negative outcomes however, as all 10 animals receiving CVVH survived, while 5 out of 10 animals in control group perished during the follow-up (*p* < 0.05, vs, CVVH treated).

**Table 1:** Hemodynamic parameters in animals at baseline and after sea water drowning

	Baseline	15 min	60 min	120 min	180 min	240 min
mABP(mmHg)						
Control	153 ± 22	127 ± 27*	124 ± 35*	125 ± 28*	118 ± 41*	113 ± 50*
CVVH	149 ± 25	128 ± 19*	126 ± 17*	132 ± 27	135 ± 15	134 ± 29
HR(beat/min)						
Control	194 ± 29	132 ± 39*	139 ± 24*	128 ± 22*	122 ± 28*	120 ± 39*
CVVH	204 ± 33	146 ± 34*	134 ± 18*	136 ± 37*	138 ± 29*	128 ± 22*
CVP(cmH _2_ O)						
Control	9.91 ± 1.43	9.03 ± 1.78	10.18 ± 1.71	10.52 ± 1.23	10.21 ± 1.43	9.37 ± 2.15
CVVH	10.21 ± 2.62	9.73 ± 3.52	10.59 ± 4.28	11.19 ± 3.98	11.03 ± 4.87	11.28 ± 4.11
Osmolarity (mOs/kg)						
Control	291 ± 11	313 ± 13*	311 ± 7*	307 ± 10*	311 ± 4*	318 ± 6*
CVVH	297 ± 15	312 ± 7*	316 ± 12*	315 ± 21*	326 ± 9* ^#^	332 ± 11*^#^

Hemodynamic parameters were monintored in animals before and 15, 50, 120, 180, and 240 minutes after involuntary intratracheal sea water infusion. All animals received mechanical ventilation after sea water infusion and then were randomly divided into control and CVVH group *p < 0.05, vs, the same group of animals before infusion (baseline); #*p* < 0.05, vs, animals in control group at the same time point

### Blood gas and acid-base balance

The levels of PaO_2_ and SaO_2_ were reduced to below 40 mmHg and less than 70 %, respectively, in animals within 15 minutes after intratracheal sea water infusion (Table 2). Mechanical ventilation via the conditions stated in the methods led to significant rescue of the levels of both markers, improvements which were further enhanced by CVVH (Table 2).

**Table 2:** Blood gas in animals at baseline and after sea water drowning

	Baseline	15 min	60 min	120 min	180 min	240 min
pH						
Control	7.35 ± 0.03	7.23 ± 0.03*	7.22 ± 0.20*	7.21 ± 0.12*	7.18 ± 0.10*	7.16 ± 0.10*
CVVH	7.37 ± 0.05	7.22 ± 0.09*	7.34 ± 0.08^#^	7.34 ± 0.06^#^	7.36 ± 0.05^#^	7.40 ± 0.08^#^
P _a_ CO _2_ (mmHg)						
Control	35.1 ± 1.21	35.17 ± 3.67	40.48 ± 7.03*	43.28 ± 5.21*	40.7 ± 5.14*	40.68 ± 5.83*
CVVH	34.5 ± 2.32	31.53 ± 7.13	33.13 ± 8.29	37.23 ± 3.18	39.33 ± 7.35	38.22 ± 8.11
P _a_ O _2_ (mmHg)						
Control	102.4 ± 4.03	33.5 ± 4.03*	58.2 ± 11.2*	61.2 ± 8.67*	61.8 ± 10.0*	65.2 ± 7.88*
CVVH	101.0 ± 2.73	36.4 ± 8.98*	61.6 ± 4.93*	68.1 ± 10.0*	76.0 ± 5.98* ^#^	86.5 ± 4.72^#^
AB (mmol/L)						
Control	21.4 ± 3.2	15.9 ± 4.0*	16.1 ± 3.6*	15.9 ± 3.8*	15.7 ± 2.6*	15.7 ± 3.9*
CVVH	22.1 ± 3.7	15.6 ± 3.4*	16.4 ± 4.1*	18.2 ± 2.4	19.9 ± 3.8^#^	21.5 ± 2.4^#^
BE (mmol/L)						
Control	–4.1 ± 0.9	–11.2 ± 5.2*	–11.1 ± 2.5*	–11.9 ± 3.8*	–12.3 ± 4.3*	–13.1 ± 3.7*
CVVH	–4.8 ± 1.5	–11.7 ± 3.8*	–9.3 ± 3.2*	–7.1 ± 2.8^#^	–5.2 ± 1.7^#^	–4.7 ± 2.9^#^
S _a_ O _2_ (%)						
Control	97.5 ± 0.92	53.9 ± 7.33*	75.6 ± 8.75*	80.9 ± 7.24*	84.4 ± 6.58*	86.6 ± 5.98*
CVVH	98.6 ± 0.58	50.2 ± 10.95*	86.6 ± 5.71	90.9 ± 4.33^#^	92.7 ± 4.62^#^	94.9 ± 3.49^#^

Blood gas was determined in arterial blood samples from animals before and 15, 50, 120, 180, and 240 minutes after involuntary intratracheal sea water infusion. All animals received mechanical ventilation after sea water infusion and then were randomly divided into control and CRRT group *p < 0.05, vs, the same group of animals before infusion (baseline); #*p* < 0.05, vs, animals in control group at the same time point

The decreases in arterial blood pH, actual bicarbonate (AB), and base excess (BE) observed suggested a state of metabolic acidosis in the animals (Table 2). While acidosis persisted over 4 hours in the control group, CVVH treatment significantly ameliorated the acidosis after 2 hours (Table 2).

### IL-8,IL-6,TNF-α, Endothelin, and SOD

**Fig. 1:**
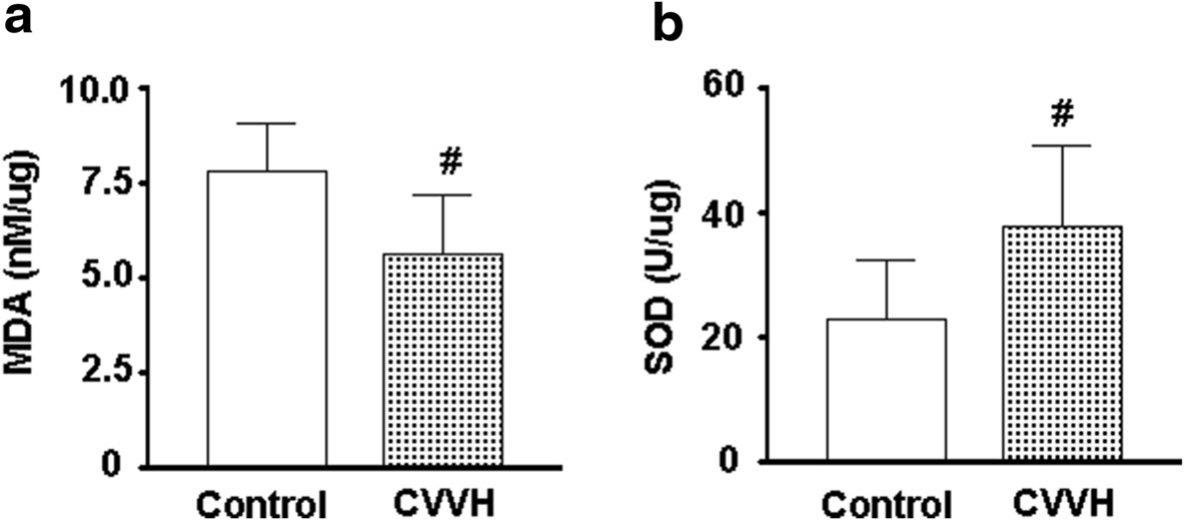
MDA and SOD activity in lungs from sea water drowning animals. Lungs were obtained from control and CVVH treated animals 4 hours and 15 minutes after involuntary intratracheal infusion of sea water. MDA levels and SOD activities were determined in lungs and the results were corrected for protein levels in the same sample. **a.** MDA levels. *p < 0.05, vs control; **b**. SOD activities. *p < 0.05, vs control

Plasma levels of IL-8, IL-6 and TNF-α were elevated in animals 15 minutes after involuntary sea water inspiration, and remained high in the control group at 4 hours (Table 3). These elevations were prevented in the CVVH group (Table 3). The levels of endothelin at sacrifice were also significantly higher in control (0.19 ± 0.11 pg/ml) than in CVVH treated animals (0.10 ± 0.04 pg/ml, *p* < 0.01, vs, the levels in control). Since the lung is the primary organ involved in sea water drowning, we also measured the levels of oxidative stress in the lungs via SOD and MDA. The SOD activity in lungs of CVVH treated animals was 47.17 ± 16.23 U/ug.pr, which was significantly higher than that in lungs of control (28.49 ± 12.09 U/ug.pr. Fig. 1). This was consistent with the observation that the levels of MDA were lower in lungs of CVVH treated animals (5.62 ± 1.58 nM/ug.pr, vs, control, 7.81 ± 1.22 nM/ug.pr, *p* < 0.05, Fig. 1).

**Table 3:** Circulating inflammatory mediators in animals at baseline and after sea water drowning

	Baseline	15 min	120 min	240 min
IL-8				
Control	81.83 ± 34.22	106.8 ± 17^*^	161.1 ± 48.6^*^	174.3 ± 59.7^*^
CVVH	87.67 ± 15.77	111.4 ± 8.3^*^	91.01 ± 17.5^#^	85.37 ± 23.6^#^
IL-6				
Control	219.4 ± 58.5	323.3 ± 96.1^*^	339.5 ± 106.5^*^	365.6 ± 119.5^*^
CVVH	212.9 ± 33.0	378.5 ± 57.7^*^	249.9 ± 48.2	221.4 ± 27.8^#^
TNF-α				
Control	53.82 ± 16.81	79.53 ± 9.48^*^	76.17 ± 8.11^*^	89.78 ± 10.5^*^
CVVH	50.34 ± 15.10	83.21 ± 8.95^*^	63.52 ± 11.3^#^	61.43 ± 17.6^#^
Endothelin				
Control	0.08 ± 0.05	N/A	N/A	0.19 ± 0.11^*^
CVVH	0.09 ± 0.04	N/A	N/A	0.10 ± 0.04^#^

Plasma IL-8, IL-6, TNF-α, and endothelin levels were from animals before and after involuntary intratracheal sea water infusion. All animals received mechanical ventilation after sea water infusion and then were randomly divided into control and CRRT group *p < 0.05, vs, the same group of animals before infusion (baseline); #*p* < 0.05,vs, animals in control group at the same time point

### Lung weight and pathology

**Fig. 2:**
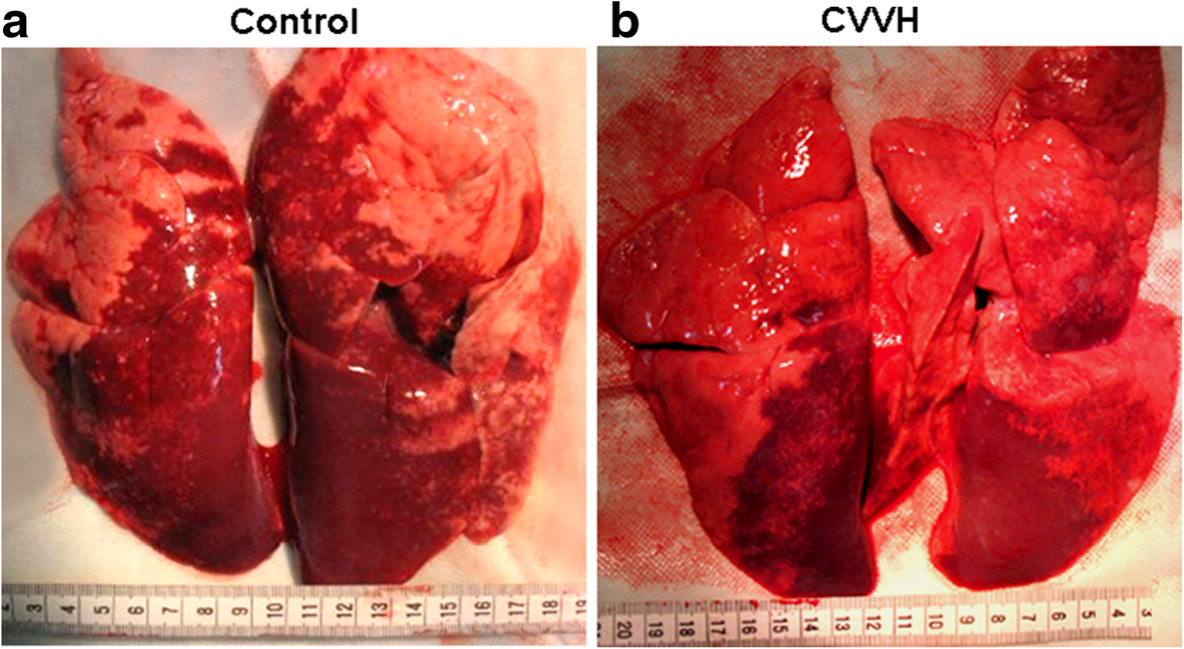
Lung tissue from sea water drowning animals. Lungs were obtained from control and CVVH treated animals 4 hours and 15 minutes after involuntary intratracheal infusion of sea water. **a.** Representative lung tissue from control group. About 2/3 of lungs appeared in dark red color, indicating severe blood congestion. **b**. Representative lung tissue from CVVH treated group. Dark red area was visibly smaller than the control

**Fig. 3:**
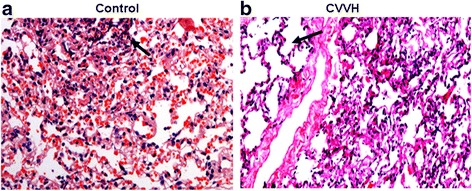
Light microscopy of lung tissue from sea water drowning animals. Lungs were obtained from control and CVVH treated animals 4 hours and 15 minutes after involuntary intratracheal infusion of sea water. Tissues were processed for hematoxylin and eosin staining. **a.** Representative lung slide from control group (×200). Red blood cells were widely present inside capillary and aveoli. Aveolar structures were distorted (arrow). **b**. Representative lung slide from CVVH treated group (×200). There was an area with well preserved aveolar structures (arrow). Red blood cells were barely seen in the tissue

At sacrifice, the wet weight of lungs from control group was 187 ± 44.8 g, which was heavier than the wet weight of lungs from CVVH treated group (152 ± 12.9 g, vs, control, *p* < 0.05). Lungs from control group appeared distended with nearly 2/3 of area showing a dark red color, suggesting severe blood congestion (Fig. 2a). Frothy and bloody exudate leaked out when the lungs from controls were cut open, further revealing the presence of pulmonary edema and blood congestion. By contrast, lungs from CVVH treated animals were mostly pinkish color (Fig. 2b), with lesser amounts of foamy exudate and blood congestion. Microscopic examination of controls from portion of lungs without severe blood congestion still showed widespread red blood cells in alveoli, interstitial space and blood vessels (Fig. 3a). Significant amounts of infiltrating immune cells were also detected amid distorted/damaged alveolar structures, and were prominent in the control group.

## Discussion

Restoration of blood-air exchange is an essential step for resuscitation from drowning, and therapies that can quickly evacuate the aspirate from alveoli are critical for rescue [10]. CVVH has been proven to be an effective modality for the treatment of pulmonary edema caused by cardiogenic diseases, but has not been previously applied to cases of drowning [11]. In this study, we demonstrate that CVVH is indeed useful for enhancing resuscitation via a number of different factors. CVVH improved blood oxygen and decreased carbon dioxide retention, significantly ameliorating the induced pulmonary edema. Drowning induced pulmonary edema is believed to derive from a complex series of biophysical interactions, including the abnormal formation of water interface on alveolar surfaces, the loss of alveolar surfactant, the disruption of the integrity of capillary membranes, and endothelial and epithelial injury. Cardiovascular dysfunction caused by severe hypoxia and hypotension and bradycardia may also contribute, and in the case of drowning, precede it, partly as a result of the mammalian diving reflex. [12] The exact mechanisms behind the sustained bradycardia observed in drowning is unclear however, especially since hypotension and hypoxia and cold shocks from immersion/aspiration should drive tachycardia [13]. Bradycardia is mechanically caused by suppressed automaticity, conduction block, or escape pacemakers and rhythms. Hypoxia and/or ischemia have been identified to a cause of bradycardia. Since our results showed significant improvements in blood oxygen and rescued hypotension after CVVH treatment helped to correct the pulmonary edema and enhance survival, without correcting bradycardia as significantly, it is not clear of the cause of bradycardia in these sea water drowning animals.

The effects of CVVH extend beyond helping restore hemodynamic stability. We observed the elevation of circulating IL-8, IL-6, and TNF-α in dogs as early as 15 minutes after sea water drowning, and remained high at 4 hours afterwards. While hypertonic saline has been shown to reduce IL-8 secretion in some cases, these increases are not entirely surprising given both the high amount of tissue damage and high-osmolality environment induced, as both have been independently shown to promote their secretion [14, 15]. IL-8, IL-6, and TNF-α are critical mediators of acute inflammatory injury that may have significant negative repercussions for both survival and afterwards. While these cytokines may be best known for the roles they play in cytokine storms in sepsis, the circulating levels of cytokines detected in the canine model of drowning might only be a little lower than that observed in sepsis [16] IL-8 and IL-6 release can cause the recruitment and polarization of pro-inflammatory immune cells while also driving increased vascular permeability, while TNF-α can induce both pro-inflammatory and apoptotic responses in a wide range of cells [17, 18]. These cytokines may also cause microglia activation and damage to the central nervous system. The levels of these pro-inflammatory cytokines have been found to be increased in patients with acute respiratory distress syndrome caused by both cardiogenic and non-cardiogenic diseases [19, 20]. Inhibition of IL-8 and its receptor CXCR2 has been found to reduce acute lung injury in animals [21]. Moreover, mice lacking IL-6 had decreased acute pancreatitis associated acute lung injury and lethality [22]. Additionally, TNF-α has been shown to act via its receptor 1 and death signaling to induce alveolar epithelial cell dysfunction in acute lung injury model [23]. Mice lacking TNF-α receptor 1 are protected from acid-aspiration induced lung injury and pulmonary edema [24]. While TNF-α may play a protective role against septic shock in chronic inflammation, it is clearly associated with negative outcomes during acute inflammation [25]. As such, the reduction in circulating IL-8, IL-6, and TNF-α as a result of CVVH is likely very beneficial. These results are similar to those obtained by other groups applying CVVH to treat a canine model of oleic acid-induced acute lung injury and to treat canine sepsis [16].

It has been previously shown that patients with acute lung injury have increased endothelin levels and the levels of endothelin reduced as patients recovered [26], with endothelin being actively involved in pulmonary edema by increasing endothelium permeability and hydrostatic pressure [18]. We found that CVVH treatment significantly decreased endothelin levels. These decreases caused by CVVH also occurred in conjunction with reductions in oxidative stress, as evidenced by lower MDA, an indicator for lipid peroxidation, and higher antioxidant SOD activity in lungs from animals treated with CVVH. It is noteworthy, however, that some of these effects of CVVH may be more indirect results following from of its correction of hemodynamic instability.

While this study was conducted with a short follow-up time (4 hours) and a rather fixed CVVH protocol for drowning resuscitation, our study clearly showed for the first time that CVVH was an effective modality in correcting hemodynamic abnormalities, reducing pulmonary edema and inflammatory lung injury, and increasing survival in a canine model of severe sea water drowning. Explorations of the precise extent to which each of these types of injuries and their resolution plays a role in survival from drowning is needed however to clarify the actual mechanisms through which CVVH was effective. For instance, a prolonged follow-up might show even better results, with the reduction in delayed tissue damage and inflammation caused by CVVH serving to help allow for faster resolution of injury, and likely protecting against additional damage to the CNS. At the same time however, extended follow up may also demonstrate that the inability of CVVH to correct bradycardia significantly diminishes its stand-alone value for therapy. Although CVVH treatment was the only additional method considered here for the sake of simplicity, future studies incorporating in core reheating, other forms of fluid resuscitation, and suppression of inflammation will be required to truly evaluate its therapeutic utility in cases of drowning.

## Conclusion

CVVH is an effective treatment method for improving survival and resolving pulmonary edema and inflammation in the case of canine seawater drowning. CVVH treatment serves to limit additional tissue damage in animals through these and other mechanisms related to hypoxia and oxidative stress, thus likely servivng to also improve later outcomes. However, CVVH is insufficient to rescue bradycardia induced in the model, and may not be able to directly help in resolving cardiac problems. Further work evaluating the efficacy of CVVH in combination with other common therapies will be necessary for understanding its therapeutic use in the context of drowning.
